# Association between body roundness index and reproductive outcomes in patients with polycystic ovary syndrome: a secondary analysis based on PCOSAct

**DOI:** 10.3389/fnut.2026.1705555

**Published:** 2026-02-05

**Authors:** Jiannan Yu, Hang Ge, Zhuwei Gao, Jiaxing Feng, Yue Gao, Jing Cong, Mengyi Zhu, Baichao Shi, Muxin Guan, Jingshu Gao, Xiaoke Wu

**Affiliations:** 1 Heilongjiang University of Chinese Medicine, Harbin, China; 2 First Affiliated Hospital of Zhejiang Chinese Medical University (Zhejiang Provincial Hospital of Traditional Chinese Medicine), Hangzhou, China; 3 First Affiliated Hospital, Heilongjiang University of Chinese Medicine, Harbin, China

**Keywords:** body roundness index, obesity, polycystic ovary syndrome, reproductive outcomes, visceral fat

## Abstract

**Background:**

Obesity, especially visceral obesity, is highly prevalent in women with polycystic ovary syndrome (PCOS) and may adversely affect fertility outcomes. Body roundness index (BRI) is an anthropometric indicator of visceral adiposity, yet evidence linking BRI to key reproductive outcomes remains limited in PCOS.

**Methods:**

This secondary analysis included 998 Chinese women from the Polycystic Ovary Syndrome Acupuncture and Clomiphene Trial (PCOSAct). Baseline BRI was calculated from waist circumference and height measurements, and reproductive outcomes were obtained after interventions. Baseline BRI was calculated from height, weight, and waist circumference and analyzed as quartiles (Q1: < 2.97, Q2: 2.97–3.78, Q3: 3.78–4.87, Q4: ≥ 4.87) and as a continuous variable. Logistic regression models estimated odds ratios (ORs) and 95% confidence intervals (CIs) for ovulation, conception, clinical pregnancy, and live birth, adjusting for interventions, age, systolic and diastolic blood pressure (SBP and DBP). Nonlinearity was assessed using restricted cubic splines (RCS), with model fit compared against linear models using likelihood ratio tests.

**Results:**

In total, 780 participants regained ovulation, 320 achieved conception, 218 attained clinical pregnancy, and 205 had a live birth. Higher BRI quartiles were associated with worse anthropometric, metabolic, and hormonal profiles at baseline. In regression analyses, higher BRI quartiles were generally associated with lower odds of reproductive outcomes, with significant trends across quartiles after adjustment. Restricted cubic spline analyses showed no evidence of nonlinearity for ovulation or conception (*P*-nonlinear = 0.951 and 0.301), but significant nonlinearity for clinical pregnancy and live birth (*P*-nonlinear = 0.016 and 0.025).

**Conclusion:**

Higher BRI was associated with poorer reproductive outcomes in women with PCOS, with evidence of nonlinear associations for clinical pregnancy and live birth. BRI may provide clinically relevant information beyond general adiposity for reproductive risk stratification in PCOS.

## 1 Introduction

Polycystic ovary syndrome (PCOS), one of the most prevalent endocrine disorders, affects 4%–21% women of reproductive age worldwide (1). PCOS is characterized by hyperandrogenemia, oligo-ovulation, and polycystic ovarian morphology, which usually results in infertility. Furthermore, a large proportion of women with PCOS suffer from obesity, accounting for 20%–80% (2). The complications resulting from obesity significantly impair both endocrine hemostasis (3) and infertility outcomes (2).

Body mass index (BMI) has been widely used as a simple anthropometric measure to assess general adiposity and is found to be associated with reproductive outcomes in PCOS (4–6). However, the predictive value remains controversial. Recent meta-analyses suggested that women with lower BMI were more likely to achieve clinical pregnancy and live birth (7, 8). Some studies reported that high BMI might not adversely affect the fertility outcomes of Chinese women with PCOS who underwent *in vitro* fertilization (IVF) (6). Increasing evidence found the body fat distribution, particularly the abdominal obesity, might have a more negative influence on clinical pregnancy rate and live birth rate (9). Despite having the same BMI, women with PCOS demonstrated greater central adiposity than women without PCOS (10, 11). A recent study, conducted in adolescence with PCOS, found that the reduction of liver and visceral fat was more effective in restoring ovulation than simply losing weight (12). Overall, excess visceral fat is more likely to worsen both metabolic function and reproductive prognosis in PCOS.

Body roundness index (BRI), an anthropometric model derived from waist circumference, height, and weight, demonstrated a superior estimate ability for visceral adiposity and central obesity compared with BMI (13, 14). In PCOS, accumulating evidence highlighted the value of BRI in predicting metabolic dysfunction, such as insulin resistance and metabolic syndrome (15–17). Several cross-sectional studies of the National Health and Nutrition Examination Survey (NHANES) revealed a significant positive correlation between BRI and infertility, which was consistent across diverse populations (18–21). Moreover, higher BRI was strongly associated with prediabetes and type 2 diabetes (22, 23), nonalcoholic fatty liver disease (24–27), cardiometabolic multimorbidity (28, 29), cardiovascular disease and all-cause mortality (28–32), and depression (33, 34). These diseases are of high incidence in women with PCOS, which bring negative effect on clinical outcomes. However, there was limit evidence revealing the association between BRI and reproductive outcomes. Body fat distribution index had more predictive ability for unexpected recurrent pregnancy loss than BMI (35). The effect of BRI on other fertility outcomes, including ovulation, conception, clinical pregnancy, and live birth, needs to be explored in PCOS.

Despite these compelling negative effects of BRI on metabolic health and pregnancy loss, its relationship with other reproductive outcomes, especially live birth, remains unclear in women with PCOS. To address this gap, this study utilized data from a large-scale, multicenter, randomized controlled trial of Chinese women with PCOS to investigate the association between BRI and key reproductive outcomes.

## 2 Methods

### 2.1 Study population and design

This study utilized data from the PCOS Acupuncture and Clomiphene Trial (PCOSAct) to investigate the relationship between BRI and reproductive outcomes in patients with PCOS. PCOSAct was a multicenter, 2 × 2 factorial randomized controlled trial conducted in China between 2012 and 2015. This trial enrolled a total of 1,000 eligible participants diagnosed with PCOS using the modified Rotterdam criteria. The participants were randomly assigned into one of four groups, each 250, and received active/sham acupuncture combined with clomiphene/placebo for a duration of 4 menstrual cycles. Both the protocol and primary results have been published (36, 37). The trial is registered at chictr.org.cn (ChiCTR-TRC-12002081) and ClinicalTrials.gov (NCT01573858), and approved by the Regional Ethics Committee of the First Affiliated Hospital of Heilongjiang University of Traditional Chinese Medicine, Harbin, China (No. 2010HZYLL-010). Written informed consent was obtained from all participants prior to enrollment.

### 2.2 Data collection

#### 2.2.1 Baseline evaluation

The baseline assessments included demographic data, anthropometric parameters, and fasting serum testing. Demographic information encompassed the age of both the patients and their partners. Height, weight, waist circumference (WC), and blood pressure (systolic blood pressure [SBP], diastolic blood pressure [DBP]), acne, and acanthosis nigricans were measured by trained research staff at each participating site following a standardized trial manual. Hirsutism was evaluated using the modified Ferriman–Gallwey scale (38). Fasting blood samples were obtained on the third day of the menstrual cycle and analyzed at the Core Laboratory of the First Affiliated Hospital of Heilongjiang University of Chinese Medicine. Specifically, the following parameters were assessed: sex hormones included estradiol (E_2_), progesterone (P), testosterone (T), free testosterone (FT), luteinizing hormone (LH), follicle-stimulating hormone (FSH), and sex hormone-binding globulin (SHBG); Glycolipid metabolism parameters assessed included fasting blood glucose (FBG), fasting insulin (FIN), total cholesterol (TC), triglycerides (TG), low-density lipoprotein (LDL), high-density lipoprotein (HDL), apolipoprotein A1 (ApoA1), and apolipoprotein B (ApoB). Additionally, related indices were calculated for clinical use. BMI was calculated as weight divided by height squared (kg/m^2^). BRI was computed according to the formula of $364.2-365.5\times \sqrt{\left[1-{\left(\mathrm{WC}/(2\pi )\right)}^{2}/{\left(0.5\times \text{height}\right)}^{2}\right]}$ established in a previous study (13). FAI was determined as T (nmol/L)/SHBG (nmol/L) × 100. Homeostatic model assessment of insulin resistance (HOMA-IR) was computed as FIN (mIU/mL) × FBG (mmol/L)/22.5. Insulin resistance (IR) was defined as HOMA-IR ≥ 2.69. Based on criteria for Chinese women, BMI < 24 kg/m^2^ was classified as normal weight, and BMI ≥ 24 kg/m^2^ was considered overweight and obese (39).

#### 2.2.2 Pregnancy outcome

Ovulation was defined as a mid-luteal serum P concentration greater than 5 ng/mL in any treatment cycle. Conception was identified by a positive urinary human chorionic gonadotropin test. Clinical pregnancy was confirmed via ultrasound detection of an intrauterine gestation with fetal cardiac activity. Live birth was defined as the delivery of a living infant at or beyond 20 weeks of gestation.

### 2.3 Statistical analyses

In descriptive analyses, participants’ baseline characteristics were stratified and compared across BRI quartiles. Normally distributed continuous variables are presented as mean ± standard deviation and compared using one-way analysis of variance (ANOVA), whereas non-normally distributed variables are expressed as median (25th, 75th percentiles) and compared using the Kruskal–Wallis test. Categorical variables are summarized as counts (percentages) and compared using chi-square tests or Fisher’s exact tests, as appropriate, when expected frequencies were below 5. Logistic regression models were used to evaluate the associations between BRI and reproductive outcomes, including ovulation rate, conception rate, clinical pregnancy rate, and live birth rate. BRI was modeled both as a categorical variable (quartiles) and as a continuous variable. For quartile analyses, odds ratios (OR) and 95% confidence intervals (CIs) were estimated using the lowest quartile as reference. Tests for linear trend were conducted by modeling the median value of each quartile as a continuous variable. Model 1 was unadjusted. Model 2 was adjusted for interventions, age, SBP and DBP. Model 3, additionally including metabolic and hormonal covariates (HOMA-IR, FBG, HDL, TG, APOA1, FT, and SHBG), was fitted as a sensitivity analysis and was presented in the Supplementary material. Notably, WC and BMI were not included in the covariate adjustment due to severe collinearity with BRI. Potential non-linear associations were evaluated using restricted cubic spline (RCS) analyses with 4 knots. To evaluate robustness to extreme BRI values, spline analyses were repeated after winsorizing BRI at the 1st and 99th percentiles (and additionally using a more stringent two-sided 5% winsorization), with all observations retained. Exploratory threshold analyses were performed using segmented (change-point) logistic regression models implemented with the package chngpt (40) (type = “segmented”), with the inflection point (change point) estimated from the data using the smooth approximation method (est.method = “smoothapprox”). Effect estimates on each side of the estimated change point were reported as OR (95% CI). Model fit was compared with that of a standard linear logistic regression model using a likelihood ratio test. Effect modification by BMI category was examined by an including interaction term between BRI and BMI category in logistic regression models. To characterize the overlap between BRI and BMI, BMI distributions across BRI quartiles were additionally visualized (boxplots) and summarized in a Supplementary material. RCS, segmented regression, and interaction analyses were conducted using the same covariate set as Model 2 (interventions, age, SBP, and DBP), unless otherwise specified. Two participants with missing BRI values were excluded prior to analysis. Missingness for all covariates included in multivariable models was low (≤5% for each variable); therefore, primary analyses were conducted using complete-case data. To evaluate whether missingness was likely to occur at random, missing-data patterns were examined, and the non-parametric MCAR test was conducted; baseline characteristics were also compared between participants with complete vs. incomplete data. As a sensitivity analysis, the main regression models were repeated in the subset of participants with complete data for all covariates included in Model3, and consistency of effect estimates was used to evaluate robustness. All analyses were conducted using R software (version 4.5.0; *R* Foundation for Statistical Computing, Vienna, Austria) and the package chngpt (40), with two-tailed *p*-values < 0.05 considered statistically significant.

## 3 Results

### 3.1 Baseline characteristics by BRI quartiles

Table 1 summarizes the baseline characteristics of participants stratified by BRI quartiles (Q1: < 2.97, Q2: 2.97–3.78, Q3: 3.78–4.87, Q4: ≥ 4.87). Increasing BRI was associated with worse anthropometric, metabolic, and hormonal profiles. Women in higher BRI quartiles showed significantly higher BMI, SBP, DBP, and acanthosis nigricans scores (all *p* < 0.001). In terms of sex hormones, higher BRI quartiles were associated with increased FT and FAI, along with decreased LH/FSH ratio, E_2_, and SHBG (all *p* < 0.01). Regarding glycolipid metabolism, significant progressive increases were observed in FIN, FBG, HOMA-IR, LDL, TC, TG, and ApoB (all *p* < 0.001), while HDL and ApoA1 decreased (*p* < 0.001). No significant differences were observed across BRI quartiles in age, paternal age, FG score, acne score, P, T, or interventions (all *p* > 0.05).

**Table 1:** Baseline characteristics of study population based on BRI quartiles.

Characteristic	Overall	BRI				*p*
		Q1 <2.97	Q2 2.97–3.78	Q3 3.78–4.87	Q4 ≥4.87	
Anthropometric measures						
Age, years	28.00 (26.00, 30.00) (998)	27.00 (26.00, 29.00) (251)	28.00 (26.00, 30.00) (248)	27.00 (26.00, 30.00) (253)	28.00 (26.00, 31.00) (246)	0.068
Paternal age, years	29.00 (27.00,32.00) (996)	29.00 (27.00, 32.00) (250)	30.00 (27.00, 32.00) (248)	29.00 (27.00, 32.00) (253)	30.00 (28.00, 32.00) (245)	0.115
BMI, kg/m^2^	23.70 (21.00, 26.72) (998)	20.08 (18.88, 21.35) (251)	22.75 (21.29, 24.05) (248)	25.36 (23.43, 27.00) (253)	28.59 (26.54, 31.62) (246)	<0.001
SBP, mmHg	110.00 (108.00, 120.00) (998)	110.00 (100.00,115.00) (251)	110.00 (105.00,120.00) (248)	114.00 (110.00,120.00) (253)	115.00 (110.00,120.00) (246)	<0.001
DBP, mmHg	75.00 (70.00, 80.00) (998)	70.00 (70.00, 80.00) (251)	73.00 (70.00, 80.00) (248)	75.00 (70.00, 80.00) (253)	80.00 (70.00, 80.00) (246)	<0.001
Clinical features						
FG score	2.00 (1.00, 5.00) (998)	2.00 (1.00, 5.00) (251)	2.00 (1.00, 5.00) (248)	3.00 (1.00, 5.00) (253)	2.00 (1.00, 5.00) (246)	0.396
Acne score	0.00 (0.00, 1.00) (998)	0.00 (0.00, 1.00) (251)	0.00 (0.00, 1.00) (248)	0.00 (0.00, 1.00) (253)	0.00 (0.00, 1.00) (246)	0.331
Acanthosis nigricans	1.00 (1.00, 1.00) (998)	1.00 (1.00, 1.00) (251)	1.00 (1.00, 1.00) (248)	1.00 (1.00, 1.00) (253)	1.00 (1.00, 2.00) (246)	<0.001
Baseline sex hormones						
LH/FSH	1.58 (1.06, 2.31) (955)	1.87 (1.19, 2.65) (243)	1.77 (1.15, 2.62) (240)	1.40 (0.95, 2.05) (238)	1.42 (0.95, 1.87) (234)	<0.001
P, nmol/L	1.73 (1.21, 2.40) (954)	1.75 (1.25, 2.51) (243)	1.73 (1.27, 2.40) (239)	1.79 (1.21, 2.41) (239)	1.64 (1.11, 2.22) (233)	0.142
E_2_, pmol/L	199.50 (158.90, 265.70) (957)	212.75 (167.90,286.05) (242)	200.20 (163.00,271.50) (241)	188.40 (148.20,250.15) (239)	195.20 (162.50,246.40) (235)	0.005
T, nmol/L	1.59 (1.20, 2.04) (958)	1.52 (1.19, 2.03) (243)	1.56 (1.21, 2.01) (241)	1.62 (1.19, 2.04) (239)	1.67 (1.20, 2.16) (235)	0.545
FT, pg/mL	2.21 (1.67, 2.83) (954)	2.05 (1.54, 2.64) (242)	2.17 (1.64, 2.75) (239)	2.22 (1.74, 2.91) (241)	2.39 (1.87, 2.96) (232)	<0.001
FAI	4.76 (2.58, 7.86) (948)	2.81 (1.91, 4.90) (240)	3.96 (2.48, 6.85) (238)	5.40 (3.33, 8.66) (237)	7.17 (4.37, 10.55) (233)	<0.001
SHBG, nmol/L	33.90 (21.80, 54.80) (953)	53.25 (36.50, 71.78) (240)	36.90 (25.08, 59.38) (238)	27.55 (20.23, 41.95) (240)	23.30 (16.90, 33.75) (235)	<0.001
Baseline fasting serum testing						
FIN, pmol/L	74.40 (47.38, 116.53) (956)	46.34 (30.93, 70.07) (242)	64.76 (43.96, 89.17) (241)	92.22 (59.37, 129.48) (238)	113.60 (78.18, 167.10) (235)	<0.001
FBG, mmol/L	5.03 (4.55, 5.53) (957)	4.86 (4.39, 5.33) (240)	4.95 (4.55, 5.45) (241)	5.14 (4.59, 5.55) (241)	5.24 (4.73, 5.86) (235)	<0.001
HOMA-IR	2.32 (1.43, 3.84) (948)	1.39 (0.91, 2.04) (238)	2.08 (1.30, 2.96) (240)	2.85 (1.90, 4.24) (236)	3.67 (2.44, 5.91) (234)	<0.001
IR	417 (44.0%) (948)	33 (13.9%)	76 (31.7%)	137 (58.1%)	171 (73.1%)	<0.001
HDL, mmol/L	1.24 (1.01, 1.48) (957)	1.40 (1.17, 1.62) (240)	1.25 (1.03, 1.53) (241)	1.17 (0.98, 1.38) (241)	1.13 (0.94, 1.36) (235)	<0.001
LDL, mmol/L	2.90 (2.37, 3.47) (956)	2.64 (2.19, 3.07) (239)	2.85 (2.34, 3.45) (240)	3.07 (2.45, 3.53) (241)	3.17 (2.52, 3.71) (236)	<0.001
TC, mmol/L	4.64 (3.98, 5.39) (956)	4.46 (3.90, 4.96) (239)	4.60 (3.94, 5.28) (240)	4.79 (4.02, 5.54) (241)	4.94 (4.22, 5.69) (236)	<0.001
TG, mmol/L	1.31 (0.93, 1.96) (957)	1.02 (0.76, 1.33) (239)	1.25 (0.91, 1.86) (240)	1.42 (1.01, 2.08) (242)	1.79 (1.16, 2.46) (236)	<0.001
APOB, g/L	0.86 (0.69, 1.06) (956)	0.73 (0.61, 0.88) (239)	0.86 (0.69, 1.01) (240)	0.92 (0.72, 1.16) (241)	1.00 (0.80, 1.21) (236)	<0.001
APOA1, g/L	1.49 (1.28, 1.70) (957)	1.58 (1.36, 1.78) (239)	1.49 (1.30, 1.75) (240)	1.44 (1.27, 1.63) (242)	1.45 (1.23, 1.64) (236)	<0.001
Intervention, *n*(%)						0.357
Acupuncture + clomiphene	250 (25.1%)	74 (29.5%)	54 (21.8%)	65 (25.7%)	57 (23.2%)	
Control acupuncture + clomiphene	249 (24.9%)	51 (20.3%)	70 (28.2%)	69 (27.3%)	59 (24.0%)	
Acupuncture + placebo	250 (25.1%)	61 (24.3%)	61 (24.6%)	66 (26.1%)	62 (25.2%)	
Control acupuncture + placebo	249 (24.9%)	65 (25.9%)	63 (25.4%)	53 (20.9%)	68 (27.6%)	

ApoA1, apolipoprotein A1; ApoB, apolipoprotein B; BMI, body mass index; BRI, body roundness index; DBP, diastolic blood pressure; E_2_, estradiol; FAI, free androgen index; FBG, fasting blood glucose; FG, Ferriman-Gallwey score; FIN, fasting insulin; FSH, follicle-stimulating hormone ratio; FT, free testosterone; HDL, high-density lipoprotein; HOMA-IR, homeostatic model assessment-insulin resistance; IR, insulin resistance; LDL, low-density lipoprotein; LH, luteinizing hormone; P, progesterone; Q, quartile; SBP, systolic blood pressure; SHBG, sex hormone-binding globulin; TC, total cholesterol; TG, triglycerides; T, total testosterone.

### 3.2 Association between BRI and reproductive outcomes

The associations between BRI and reproductive outcomes (ovulation, conception, clinical pregnancy, and live birth) are summarized in Table 2. In quartile analyses, higher BRI quartiles were generally associated with lower odds of ovulation, conception, clinical pregnancy, and live birth, and the overall trends across quartiles were statistically significant in both unadjusted and adjusted models (*P* for trend < 0.05). Similar results were found in the analysis using a per-unit increase: higher BRI tended to have worse reproductive outcomes (all *p* < 0.05). However, there was no statistical difference between lower BRI and conception (OR: 0.92, 95% CI: 0.83–1.02) as well as clinical pregnancy (OR: 0.89, 95% CI: 0.79–1.00).

**Table 2:** BRI by quartiles and association with reproductive outcomes.

BRI	Ovulation, *n* (%)				Conception, *n* (%)			
	No	Yes	Crude^a^ OR (95% CI), *p*	Adjusted^b^ OR (95% CI), *p*	No	Yes	Crude^a^ OR (95% CI), *p*	Adjusted^b^ OR (95% CI), *p*
Quartile								
Q1	41 (16.3)	210 (83.7)	Reference	Reference	159 (63.3)	92 (36.7)	Reference	Reference
Q2	48 (19.4)	200 (80.6)	0.81 (0.51–1.29), 0.379	0.82 (0.51–1.35), 0.451	166 (66.9)	82 (33.1)	0.85 (0.59–1.23), 0.400	0.91 (0.62–1.33), 0.613
Q3	51 (20.2)	202 (79.8)	0.77 (0.49–1.22), 0.267	0.80 (0.49–1.30), 0.365	171 (67.6)	82 (32.4)	0.83 (0.57–1.20), 0.317	0.83 (0.56–1.22), 0.340
Q4	78 (31.7)	168 (68.3)	0.42 (0.27–0.64), <0.001	0.43 (0.27–0.69), <0.001	182 (74.0)	64 (26.0)	0.61 (0.41–0.89), 0.011	0.65 (0.43–0.98), 0.038
*P* for trend			<0.001	<0.001			0.012	0.032
Per 1 unit increase			0.80 (0.73–0.89), <0.001	0.81 (0.72–0.90), <0.001			0.90 (0.82–0.99), 0.040	0.92 (0.83–1.02), 0.105

BRI	Clinical pregnancy, *n* (%)				Live birth, *n* (%)			
	No	Yes	Crude^a^ OR (95% CI), *p*	Adjusted^b^ OR (95% CI), *p*	No	Yes	Crude^a^ OR (95% CI), *p*	Adjusted^b^ OR (95% CI), *p*
Quartile								
Q1	184 (73.3)	67 (26.7)	Reference	Reference	186 (74.1)	65 (25.9)	Reference	Reference
Q2	186 (75.0)	62 (25.0)	0.92 (0.61–1.37), 0.666	0.98 (0.65–1.48), 0.912	190 (76.6)	58 (23.4)	0.87 (0.58–1.31), 0.516	0.96 (0.63–1.45), 0.830
Q3	205 (81.0)	48 (19.0)	0.64 (0.42–0.98), 0.040	0.65 (0.42–1.01), 0.056	208 (82.2)	45 (17.8)	0.62 (0.40–0.95), 0.028	0.65 (0.41–1.01), 0.060
Q4	205 (83.3)	41 (16.7)	0.55 (0.35–0.85), 0.007	0.60 (0.38–0.94), 0.027	209 (85.0)	37 (15.0)	0.51 (0.32–0.79), 0.003	0.57 (0.36–0.91), 0.020
*P* for trend			0.003	0.009			0.001	0.008
Per 1 unit increase			0.87 (0.78–0.97), 0.017	0.89 (0.79–1.00), 0.054			0.85 (0.76–0.95), 0.006	0.88 (0.78–0.99), 0.035

a Non-adjusted.

b All adjusted for interventions, age, SBP, and DBP.

### 3.3 Detection of nonlinear relationships

**Figure 1:**
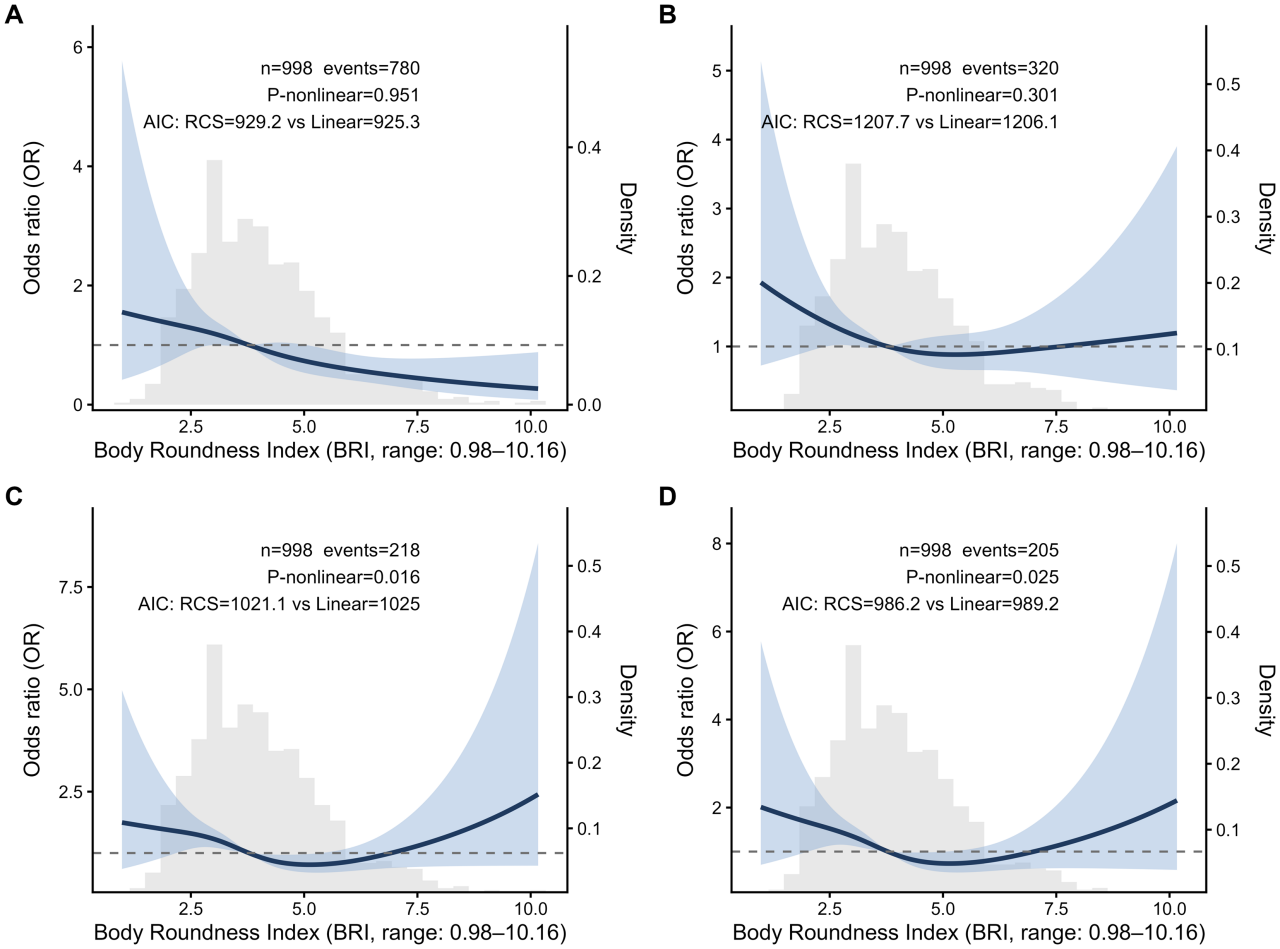
Non-linear relationship between BRI and ovulation **(A)**, conception **(B)**, clinical pregnancy **(C)**, and live birth **(D)**. All adjusted for interventions, age, SBP, and DBP. The solid line represents the estimated OR relative to the median BRI, and the dashed line indicates OR = 1. The distribution of BRI values is shown as a density plot. Model fit between nonlinear and linear models was compared using Akaike’s information criterion. Results are presented across the observed range of BRI values.

To better characterize the relationship between BRI and reproductive outcomes, RCS analyses based on logistic regression models were conducted (Figure 1). For ovulation and conception, no significant nonlinear relationships were observed (*P*-nonlinear = 0.951 for ovulation, *P*-nonlinear = 0.301 for conception). In contrast, significant nonlinear associations were observed for clinical pregnancy rate and live birth rate (*P*-nonlinear = 0.016 and 0.025, respectively). These suggested that the inverse associations were not constant across the BRI distribution and appeared strongest in the low-to-moderate range, with attenuation and a possible upturn at higher BRI values. At higher BRI values, the 95% CI widened, and the estimated odds exceeded unity in portions of the right tail, indicating increased uncertainty and reduced precision of the tail estimates.

Exploratory threshold analyses using a segmented logistic regression model identified inflection points at 5.28 (clinical pregnancy) and 5.20 (live birth) (Supplementary Table S6), which occur in the higher BRI range of Q4. Below these thresholds, each 1-unit increase in BRI was associated with 25% lower odds of clinical pregnancy (OR = 0.75, 95% CI: 0.63–0.88) and 26% lower odds of live birth (OR = 0.74, 95% CI: 0.62–0.88). Above the thresholds, the association with clinical pregnancy shifted in the positive direction (OR = 1.33, 95% CI: 1.01–1.73), but not significantly with live birth (OR = 1.28, 95% CI: 0.96–1.68). Model comparisons confirmed that the nonlinear models provided significantly better fit than linear models (likelihood ratio test *p* < 0.01 for both).

### 3.4 Sensitivity analyses

Using the non-parametric MCAR test, we found no statistical evidence against the assumption that data were missing completely at random (*p* = 0.145). In addition, baseline characteristics were comparable between participants with complete and incomplete data, suggesting that missingness was unlikely to be strongly related to key exposure or confounding variables (Supplementary Table S2). Primary models were repeated among participants with complete data for all covariates required for Model 3 (*N* = 923), and results were materially unchanged (Supplementary Table S5 and Supplementary Figure S3). Spline patterns were robust to mild winsorization (1%), while tail estimates were sensitive under heavier winsorization (5%), supporting cautious interpretation at extreme BRI values (Supplementary Figures S4, S5). In BMI-stratified analyses, there was no evidence of effect modification by BMI category (all P-interaction >0.05) (Supplementary Table S7).

## 4 Discussion

In this study, we systematically investigated the association between BRI and reproductive outcomes in Chinese women with PCOS. The principal finding was that higher BRI was significantly associated with a reduced ovulation rate. A negative association between BRI and conception rate was also observed, although this relationship was attenuated after adjustment for potential confounders. Moreover, BRI showed a nonlinear association with clinical pregnancy and live birth rates. In the low to moderate BRI range, increasing BRI was consistently associated with both clinical pregnancy and live birth. In the higher BRI range, where data were limited, the observed increase in clinical pregnancy rate should be interpreted with caution.

The existing evidence demonstrates that obesity, particularly visceral obesity, is a clear risk factor for adverse pregnancy outcomes. A retrospective study of 22,043 first frozen embryo transfer (FET) cycles among women under a freeze-all policy showed that obesity (BMI ≥ 27.5 kg/m^2^) was associated with low pregnancy and live birth rates. This negative effect may be attributable to impaired endometrial receptivity (41). A meta-analysis of 21 studies also confirmed this finding. Compared with women with normal weight (BMI 18.5–24.9 kg/m^2^), women with obesity (BMI ≥ 30 kg/m^2^) had a lower live birth rate after IVF (RR 0.85, 95% CI 0.82–0.87). Outcomes were even worse in women with PCOS (5). It is worth noting that BMI serves as a conventional indicator of obesity, reflecting overall weight status but not distinguishing between different types of body fat distribution. Visceral fat area (VFA) is a more direct measure of body fat distribution. Studies using VFA have reached similar conclusions. A retrospective clinical study involving 1,510 participants who underwent FET found that, even among those with a BMI < 24 kg/m^2^, high VFA was significantly associated with lower clinical pregnancy rates and live birth rates (9). This suggests that body fat distribution, as assessed by VFA, may be more consistently correlated with reproductive outcomes than general adiposity measured by BMI alone. However, VFA usually requires CT or MRI. These tests are expensive and not widely available in routine practice. Therefore, there is a strong need for a simple index that can reflect visceral obesity.

BRI is a novel anthropometric index that integrates waist circumference and height to predict visceral adipose tissue (13). It has demonstrated superior capability over BMI in evaluating obesity-related health risks and adipose tissue distribution (34). In reproductive health, early studies have also suggested that BRI may be useful. Analyses based on NHANES 2013–2018 data have revealed a positive, non-linear relationship between BRI and the risk of female infertility (20, 21). In addition, Kiremitli T and colleagues reported that women with unexplained recurrent miscarriage had a higher BRI (4.4 ± 1.7) than controls (3.9 ± 1.5). However, most existing work focuses on infertility or miscarriage risk (35). Evidence is still limited on whether BRI affects specific reproductive outcomes in women with PCOS, such as ovulation, pregnancy rate, and live birth. In general, our study found that BRI was negatively associated with reproductive outcomes in women with PCOS. This finding is consistent with prior studies. Studies by Ibáñez L demonstrated that reduction in hepato-visceral fat content was strongly associated with higher post-treatment ovulation rates in women with PCOS (12, 42). Similarly, another study found that early and consistent loss of intra-abdominal fat (IAF) was associated with the resumption of ovulation (43). However, a cross-sectional study reported that subcutaneous abdominal fat, but not IAF, was associated with anovulation in women with obesity and infertility (no specific indication of PCOS), even after matching for similar BMI (44). The differences may relate to patient features, study design, and how fat was measured; further studies using imaging-based measures and well-defined PCOS phenotypes are needed to clarify these relationships.

Given that we have further observed a nonlinear relationship between BRI and clinical pregnancy and live birth, it is equally important to explore the possible threshold of BRI and its clinical significance. NHANES III included 3,158 non-pregnant and non-lactating women aged ≥18 years, and the BRI range was 1.27–14.84 (13). In our study, BRI ranged from 0.98 to 10.16. This difference may reflect differences in ethnicity and population characteristics. In addition, current research has not yet established a universally accepted reference range for BRI. A large-scale analysis of 32,995 participants from the NHANES (1999–2018) suggested a potential optimal range, finding that individuals with a BRI between 4.5 and 5.5 exhibited relatively better health status and lower mortality risk (34). However, it should be noted that this population-based range was derived from general health and mortality endpoints; therefore may not be directly transferable to reproductive endpoints in women with PCOS.

We observed that within the low-to-moderate BRI range (<5.28), increasing BRI was linked to poor reproductive outcomes. This suggested that women with PCOS may have an unfavorable pattern of central fat and metabolic status even when BRI is at a relatively low level. Previous studies also reported that non-obese women with PCOS can have more visceral fat and higher inflammatory markers than healthy controls (11). This excess visceral fat drives a state of chronic low-grade inflammation, primarily mediated by the release of pro-inflammatory cytokines from adipose tissue. Acting in concert with IR and hyperandrogenemia, this inflammatory environment disrupts the hypothalamic-pituitary-ovarian (HPO) axis. The convergence of these pathologies exacerbates both metabolic and reproductive dysfunction in PCOS (3). Notably, weight loss interventions, particularly those reducing abdominal fat, have been associated with restored ovulation and improved menstrual cyclicity in affected individuals (45). Consequently, we hypothesize that strategies aimed at controlling visceral fat accumulation and limiting BRI elevation may improve pregnancy outcomes in patients with PCOS; however, further prospective and interventional studies are needed to confirm this.

Notably, when BRI is in the higher range, clinical pregnancy rates show a positive correlation with the increase in BRI. However, current statistical and clinical evidence does not support viewing this pattern as a true biological benefit. BRI distribution in our study was right-skewed, and the number of participants in the high-BRI range was small. This led to wide confidence intervals, and the curve was more likely to be driven by influential observations. The sensitivity analyses supported this concern. After 1% winsorization, the spline curve was similar to that from the full dataset. In contrast, 5% winsorization shifted the apparent turning point to the right and weakened the upward trend. This suggests that extreme values in a sparse data range may create a visual modeling artifact. Moreover, the positive slope above the turning point for live birth was not statistically significant (*p* = 0.073), providing no evidence of a beneficial association. From a biological perspective, severe obesity is usually linked to poorer oocyte quality, reduced endometrial receptivity, and a higher risk of miscarriage (46). Therefore, a strong protective effect at a very high level of BRI lacks a plausible mechanism. Overall, the main purpose of our segmented or spline analyses was to show the risk gradient and possible threshold effects within the common range of BRI. Any positive association in the right tail should be treated as exploratory rather than conclusive. Future studies with larger, multi-center studies and sufficient numbers of women in the high-BRI range are needed to validate whether the observed positive correlation is reproducible or merely reflects sparse-data instability.

The main strength of this study lies in its large sample size and data from a prospective randomized controlled trial involving Chinese women, with adjustments for several potential confounders. However, several limitations should be acknowledged. First, as an observational study, it is not possible to establish a causal relationship between BRI and reproductive outcomes in women with PCOS. Second, the study population consisted solely of women with PCOS, lacking a non-PCOS control group. Future studies that include non-PCOS participants would help further clarify the role of BRI in reproductive outcomes. Third, because this study was conducted exclusively in a Chinese population, it may limit generalizability to other ethnic groups and clinical contexts. Ethnic differences in body composition and variability in PCOS phenotypes and metabolic profiles across populations may influence the relationship between BRI and reproductive outcomes. Therefore, our findings require further validation in multi-ethnic cohorts and diverse clinical settings.

## 5 Conclusion

This study systematically evaluated the associations between BRI and reproductive outcomes in Chinese women with PCOS. Higher BRI was significantly associated with a lower ovulation rate. A negative association with conception was also observed. In addition, BRI showed a non-linear relationship with clinical pregnancy and live birth.

As a simple and accessible anthropometric measure, BRI may provide information beyond BMI and help identify women at higher risk of poor reproductive outcomes. Future multi-center studies in different populations are needed to validate these findings, further clarify the application threshold of BRI related to reproductive outcomes, and test whether interventions that improve central fat distribution can translate into better pregnancy and live birth outcomes.

## Data Availability

The raw data supporting the conclusions of this article will be made available by the corresponding authors, upon reasonable request.
