# Supplementary material for: Association between body roundness index and reproductive outcomes in patients with polycystic ovary syndrome: a secondary analysis based on PCOSAct
Source: Front Nutr. 2026 Feb 5;13:1705555. doi: 10.3389/fnut.2026.1705555 (PMC12916401; doi:10.3389/fnut.2026.1705555)

**Supplemental Material**

**eFigure 1. Timeline diagram of baseline BRI assessment and subsequent reproductive outcomes**


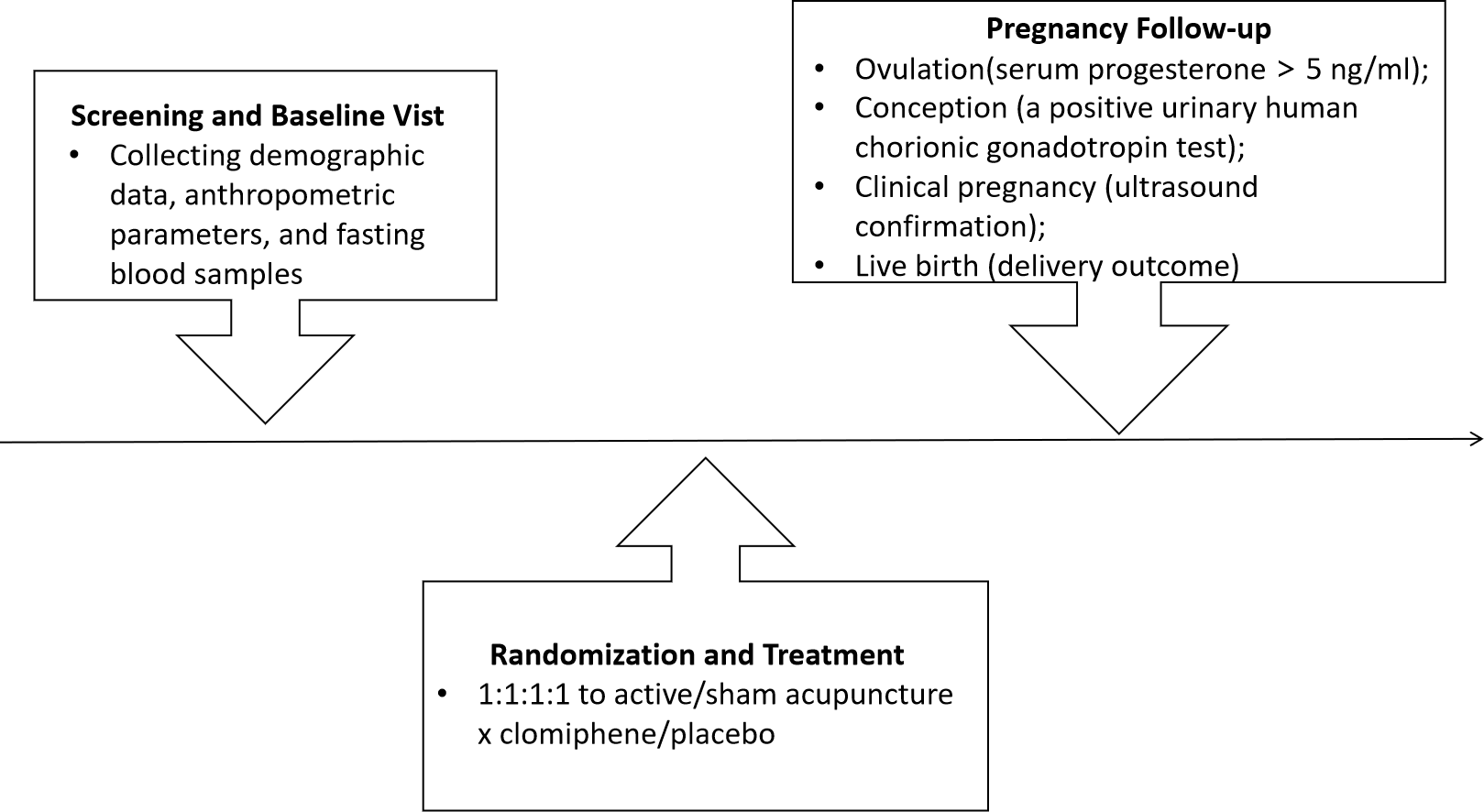


**eFigure 2. Study flowchart including missing-data exclusions**


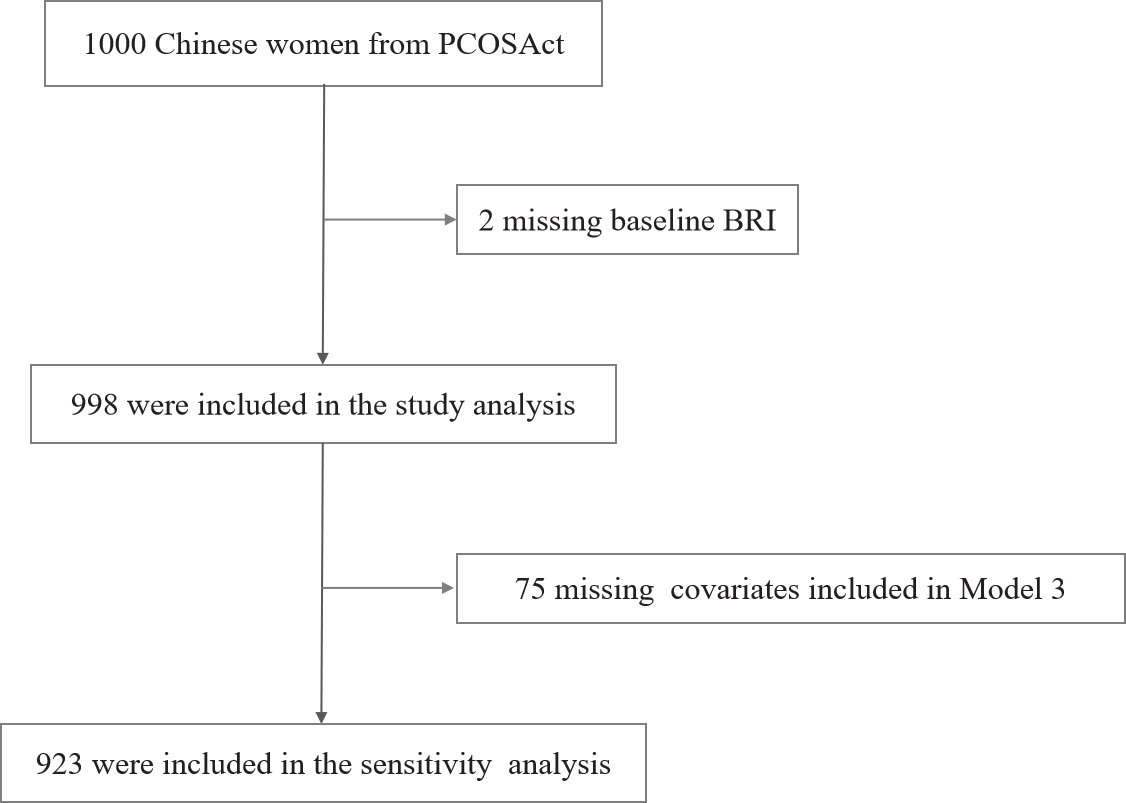


**eTable 1. Missing rate of variables.**

| Variables | total | Missing data(n, percent%) |
| --- | --- | --- |
| Interventions | 998 | 0,0 |
| age | 998 | 0,0 |
| SBP | 998 | 0,0 |
| DBP | 998 | 0,0 |
| BRI | 998 | 0,0 |
| HOMA-IR | 998 | 50,5.01 |
| FBG | 998 | 41,4.11 |
| HDL | 998 | 41,4.11 |
| TG | 998 | 41,4.11 |
| APOA1 | 998 | 41,4.11 |
| FT | 998 | 44,4.41 |
| SHBG | 998 | 45,4.51 |
| Paternal age | 998 | 2,0.2 |
| BMI | 998 | 0,0 |
| FG score | 998 | 0,0 |
| Acne score | 998 | 0,0 |
| Acanthosis nigricans | 998 | 0,0 |
| LH/FSH | 998 | 43,4.31 |
| P | 998 | 44,4.41 |
| E_2_ | 998 | 41,4.11 |
| T | 998 | 40,4.01 |
| FAI | 998 | 50,5.01 |
| FIN | 998 | 42,4.21 |
| LDL | 998 | 42,4.21 |
| TC | 998 | 42,4.21 |
| APOB | 998 | 42,4.21 |
| IR | 998 | 50,5.01 |
| Ovulation | 998 | 0,0 |
| Conception | 998 | 0,0 |
| Clinical pregnancy | 998 | 0,0 |
| Live birth | 998 | 0,0 |

**eTable 2. Baseline characteristics according to missing data status (based on Model 3, complete-case vs incomplete)**

| Variable | Complete  N=923 | Incomplete  N=75 | *P* |
| --- | --- | --- | --- |
| Interventions | 2.00 [1.00, 3.00] | 3.00 [2.00, 4.00] | 0.480 |
| age | 28.00 [26.00, 30.00] | 27.00 [25.00, 30.00] | 0.576 |
| SBP | 110.00 [108.00, 120.00] | 110.00 [107.50, 120.00] | 0.818 |
| DBP | 75.00 [70.00, 80.00] | 75.00 [70.00, 80.00] | 0.761 |
| BRI | 3.78 [2.97, 4.87] | 4.01 [2.96, 4.87] | 0.850 |
| HOMA-IR | 2.34 [1.43, 3.83] | 2.12 [1.54, 5.63] | 0.652 |
| FBG | 5.02 [4.54, 5.52] | 5.20 [4.56, 5.75] | 0.358 |
| HDL | 1.24 [1.01, 1.49] | 1.16 [0.91, 1.38] | 0.198 |
| TG | 1.30 [0.93, 1.96] | 1.46 [0.77, 2.13] | 0.717 |
| APOA1 | 1.49 [1.28, 1.71] | 1.46 [1.25, 1.64] | 0.540 |
| FT | 2.21 [1.68, 2.83] | 2.04 [1.64, 2.77] | 0.572 |
| SHBG | 33.90 [21.80, 54.80] | 31.25 [16.08, 44.50] | 0.312 |
| Paternal age | 30.00 [27.00, 32.00] | 29.00 [26.00, 32.00] | 0.232 |
| BMI | 23.63 [21.02, 26.62] | 24.74 [20.71, 28.06] | 0.207 |
| FG score | 2.00 [1.00, 5.00] | 2.00 [1.00, 5.00] | 0.623 |
| Acne score | 0.00 [0.00, 1.00] | 0.00 [0.00, 1.00] | 0.219 |
| Acanthosis nigricans | 1.00 [1.00, 1.00] | 1.00 [1.00, 2.00] | 0.061 |
| LH/FSH | 1.60 [1.08, 2.33] | 1.19 [0.75, 2.05] | 0.088 |
| P | 1.74 [1.21, 2.40] | 1.47 [1.26, 2.25] | 0.549 |
| E₂ | 198.90 [158.95, 266.15] | 210.05 [157.50, 245.38] | 0.909 |
| T | 1.60 [1.20, 2.04] | 1.42 [1.08, 1.84] | 0.247 |
| FAI | 4.78 [2.58, 7.81] | 4.10 [2.65, 8.25] | 0.986 |
| FIN | 74.01 [47.38, 116.15] | 75.50 [47.89, 123.80] | 0.341 |
| LDL | 2.90 [2.38, 3.48] | 2.81 [2.06, 3.29] | 0.176 |
| TC | 4.64 [3.98, 5.39] | 4.65 [3.76, 5.38] | 0.859 |
| APOB | 0.86 [0.69, 1.06] | 0.88 [0.65, 1.13] | 0.640 |
| IR | 0.00 [0.00, 1.00] | 0.00 [0.00, 1.00] | 0.684 |
| ovulation | 1.00 [1.00, 1.00] | 1.00 [0.00, 1.00] | 0.294 |
| conception | 0.00 [0.00, 1.00] | 0.00 [0.00, 1.00] | 0.788 |
| clinical_pregnancy | 0.00 [0.00, 0.00] | 0.00 [0.00, 0.00] | 0.688 |
| live_birth | 0.00 [0.00, 0.00] | 0.00 [0.00, 0.00] | 0.676 |

Participants were classified as having complete or incomplete data based on the availability of all covariates included in Model 3. Continuous variables are presented as mean ± standard deviation or median (interquartile range), as appropriate, and categorical variables as number (percentage). *P*-values represent comparisons between groups and are provided for descriptive purposes only. Using the non-parametric MCAR test, we found no statistical evidence against the assumption that data were missing completely at random (*P* = 0.145).

**eTable 3. Continuous BRI and reproductive outcomes: odds ratios per 1-unit and per 1-SD increase in BRI (N=998).**

| Outcome | Model 1 | Model 2 |
| --- | --- | --- |
| Ovulation |  |  |
| Per 1 unit increase | 0.80 (0.73-0.89) | 0.81 (0.72-0.90) |
| Per 1-SD increase | 0.73 (0.63-0.84) | 0.74 (0.63-0.86) |
| Conception |  |  |
| Per 1 unit increase | 0.90 (0.82-0.99) | 0.92 (0.83-1.02) |
| Per 1-SD increase | 0.87 (0.75-0.99) | 0.89 (0.77-1.02) |
| Clinical pregnancy |  |  |
| Per 1 unit increase | 0.87 (0.78-0.97) | 0.89 (0.79-1.00) |
| Per 1-SD increase | 0.82 (0.70-0.96) | 0.85 (0.72-1.00) |
| Live birth |  |  |
| Per 1 unit increase | 0.85 (0.76-0.95) | 0.88 (0.78-0.99) |
| Per 1-SD increase | 0.79 (0.67-0.93) | 0.83 (0.70-0.98) |

**eTable 4. Table 2 with Exact P Values**

| BRI | Ovulation, n (%) | | | | Conception, n (%) | | | |
| --- | --- | --- | --- | --- | --- | --- | --- | --- |
|  | No | Yes | Crude^a^ OR (95% CI), *P* | Adjusted^b^ OR (95% CI), *P* | No | Yes | Crude^a^ OR (95% CI), *P* | Adjusted^b^ OR (95% CI), *P* |
| Quartile |  |  |  |  |  |  |  |  |
| Q1 | 41 (16.3) | 210 (83.7) | reference | reference | 159 (63.3) | 92 (36.7) | reference | reference |
| Q2 | 48 (19.4) | 200 (80.6) | 0.81 (0.51-1.29), 0.379 | 0.82 (0.51-1.35), 0.451 | 166 (66.9) | 82 (33.1) | 0.85 (0.59-1.23), 0.400 | 0.91 (0.62-1.33), 0.613 |
| Q3 | 51 (20.2) | 202 (79.8) | 0.77 (0.49-1.22), 0.267 | 0.80 (0.49-1.30), 0.365 | 171 (67.6) | 82 (32.4) | 0.83 (0.57-1.20), 0.317 | 0.83 (0.56-1.22), 0.340 |
| Q4 | 78 (31.7) | 168 (68.3) | 0.42 (0.27-0.64), 0.00008 | 0.43 (0.27-0.69), 0.00046 | 182 (74.0) | 64 (26.0) | 0.61 (0.41-0.89), 0.011 | 0.65 (0.43-0.98), 0.038 |
| *P* for trend |  | | 0.000028 | 0.00019 |  | | 0.012 | 0.032 |
| Per 1 unit increase |  | | 0.80 (0.73-0.89), 0.00002 | 0.81 (0.72-0.90), 0.00015 |  | | 0.90 (0.82-0.99), 0.040 | 0.92 (0.83-1.02), 0.105 |
| BRI | Clinical pregnancy, n (%) | | | | Live birth, n (%) | | | |
|  | No | Yes | Crude^a^ OR (95% CI), *P* | Adjusted^b^ OR (95% CI), *P* | No | Yes | Crude^a^ OR (95% CI), *P* | Adjusted^b^ OR (95% CI), *P* |
| Quartile |  |  |  |  |  |  |  |  |
| Q1 | 184 (73.3) | 67 (26.7) | reference | reference | 186 (74.1) | 65 (25.9) | reference | reference |
| Q2 | 186 (75.0) | 62 (25.0) | 0.92 (0.61-1.37), 0.666 | 0.98 (0.65-1.48), 0.912 | 190 (76.6) | 58 (23.4) | 0.87 (0.58-1.31), 0.516 | 0.96 (0.63-1.45), 0.830 |
| Q3 | 205 (81.0) | 48 (19.0) | 0.64 (0.42-0.98), 0.040 | 0.65 (0.42-1.01), 0.056 | 208 (82.2) | 45 (17.8) | 0.62 (0.40-0.95), 0.028 | 0.65 (0.41-1.01), 0.060 |
| Q4 | 205 (83.3) | 41 (16.7) | 0.55 (0.35-0.85), 0.007 | 0.60 (0.38-0.94), 0.027 | 209 (85.0) | 37 (15.0) | 0.51 (0.32-0.79), 0.003 | 0.57 (0.36-0.91), 0.020 |
| *P* for trend |  | | 0.003 | 0.009 |  | | 0.001 | 0.008 |
| Per 1 unit increase |  | | 0.87 (0.78-0.97), 0.017 | 0.89 (0.79-1.00), 0.054 |  | | 0.85 (0.76-0.95), 0.006 | 0.88 (0.78-0.99), 0.035 |

**eTable 5. Sensitivity Analysis: Association between BRI and** **reproductive outcomes (N=923).**

| **Exposure** | **N=923** | | |
| --- | --- | --- | --- |
|  | **Model 1** | **Model 2** | **Model3** |
| **Ovulation** |  |  |  |
| BRI (quartile) |  |  |  |
| Q1 | Reference | Reference | Reference |
| Q2 | 0.87 (0.54–1.40), P=0.574 | 0.89 (0.53–1.47), P=0.636 | 1.00 (0.59–1.70), P=0.992 |
| Q3 | 0.84 (0.53–1.36), P=0.483 | 0.88 (0.52–1.48), P=0.625 | 1.08 (0.62–1.88), P=0.799 |
| Q4 | 0.46 (0.29–0.71), P=<0.001 | 0.47 (0.29–0.78), P=0.003 | 0.61 (0.35–1.07), P=0.084 |
| P for trend | <0.001 | 0.001 | 0.041 |
| Per 1 unit increase | 0.81 (0.73–0.90), P=<0.001 | 0.82 (0.73–0.92), P=<0.001 | 0.86 (0.76–0.99), P=0.031 |
| **Conception** |  |  |  |
| BRI (quartile) |  |  |  |
| Q1 | Reference | Reference | Reference |
| Q2 | 0.88 (0.60–1.29), P=0.514 | 0.88 (0.59–1.32), P=0.533 | 0.96 (0.64–1.46), P=0.860 |
| Q3 | 0.89 (0.60–1.30), P=0.535 | 0.85 (0.56–1.28), P=0.434 | 0.99 (0.64–1.53), P=0.977 |
| Q4 | 0.64 (0.43–0.96), P=0.029 | 0.67 (0.44–1.02), P=0.064 | 0.85 (0.53–1.36), P=0.486 |
| P for trend | 0.032 | 0.064 | 0.497 |
| Per 1 unit increase | 0.92 (0.83–1.01), P=0.078 | 0.93 (0.83–1.03), P=0.151 | 0.99 (0.88–1.11), P=0.852 |
| **Clinical pregnancy** |  |  |  |
| BRI (quartile) |  |  |  |
| Q1 | Reference | Reference | Reference |
| Q2 | 0.90 (0.60–1.37), P=0.632 | 0.93 (0.60–1.43), P=0.730 | 1.05 (0.67–1.64), P=0.833 |
| Q3 | 0.66 (0.42–1.02), P=0.058 | 0.65 (0.41–1.04), P=0.071 | 0.77 (0.47–1.26), P=0.296 |
| Q4 | 0.55 (0.35–0.86), P=0.009 | 0.59 (0.37–0.95), P=0.031 | 0.77 (0.45–1.32), P=0.347 |
| P for trend | 0.004 | 0.014 | 0.225 |
| Per 1 unit increase | 0.87 (0.78–0.98), P=0.018 | 0.89 (0.79–1.00), P=0.057 | 0.96 (0.84–1.10), P=0.566 |
| **Live birth** |  |  |  |
| BRI (quartile) |  |  |  |
| Q1 | Reference | Reference | Reference |
| Q2 | 0.84 (0.55–1.28), P=0.421 | 0.88 (0.57–1.37), P=0.579 | 0.99 (0.63–1.56), P=0.983 |
| Q3 | 0.62 (0.40–0.96), P=0.033 | 0.64 (0.40–1.02), P=0.061 | 0.77 (0.47–1.26), P=0.290 |
| Q4 | 0.49 (0.31–0.78), P=0.003 | 0.55 (0.34–0.90), P=0.018 | 0.74 (0.43–1.29), P=0.289 |
| P for trend | 0.001 | 0.009 | 0.205 |
| Per 1 unit increase | 0.84 (0.75–0.95), P=0.005 | 0.87 (0.77–0.99), P=0.030 | 0.95 (0.82–1.09), P=0.456 |

Model1: Non-adjusted.

Model2: Adjusted for interventions, age, SBP, and DBP.

Model3: Adjusted for interventions, age, SBP, DBP, HOMA-IR, FBG, HDL, TG, APOA1, FT, SHBG.

**eFigure 3. Sensitivity Analysis: RCS Curves (N = 923; Model 3)**


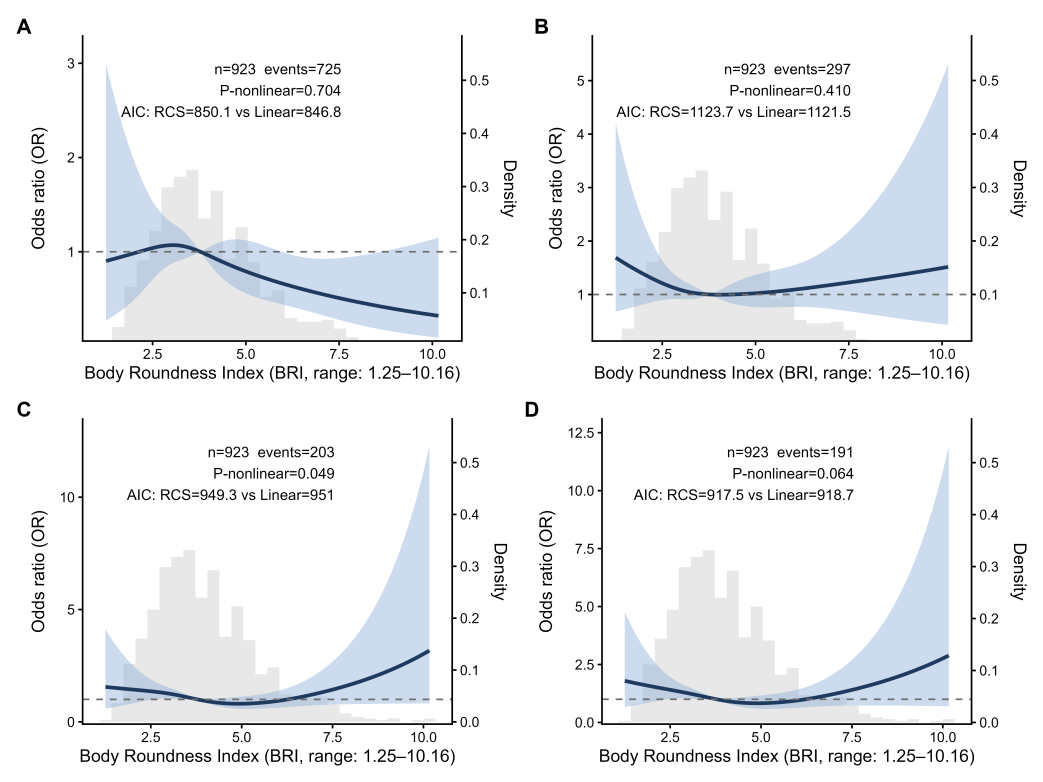


Adjusted for interventions, age, SBP, DBP, HOMA-IR, FBG, HDL, TG, APOA1, FT, SHBG.

**eFigure 4. Sensitivity Analysis: RCS Curves (1% Winsorized; N = 998; Model 2)**


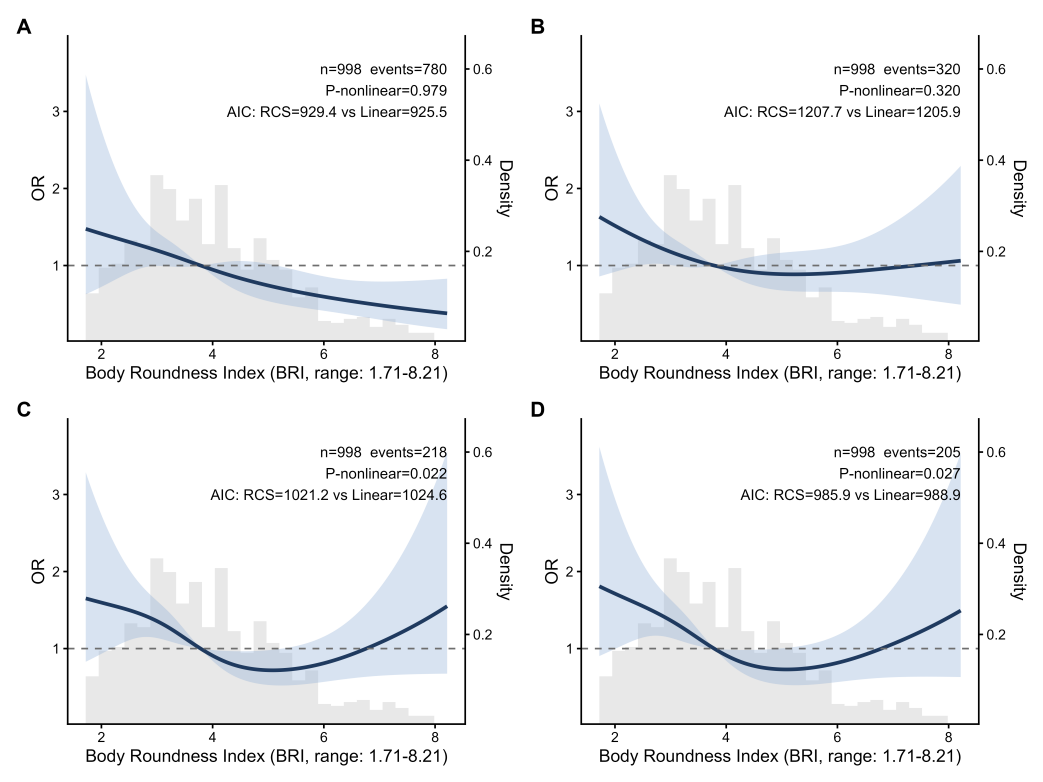


All adjusted for interventions, age, SBP, and DBP. To reduce the influence of extreme values, BRI was winsorized at the 1st and 99th percentiles prior to restricted cubic spline analyses. Values below the 1st percentile and above the 99th percentile were replaced with the corresponding percentile values, while all observations were retained.

**eFigure 5. Sensitivity Analysis: RCS Curves (5% Winsorized; N = 998; Model 2)**


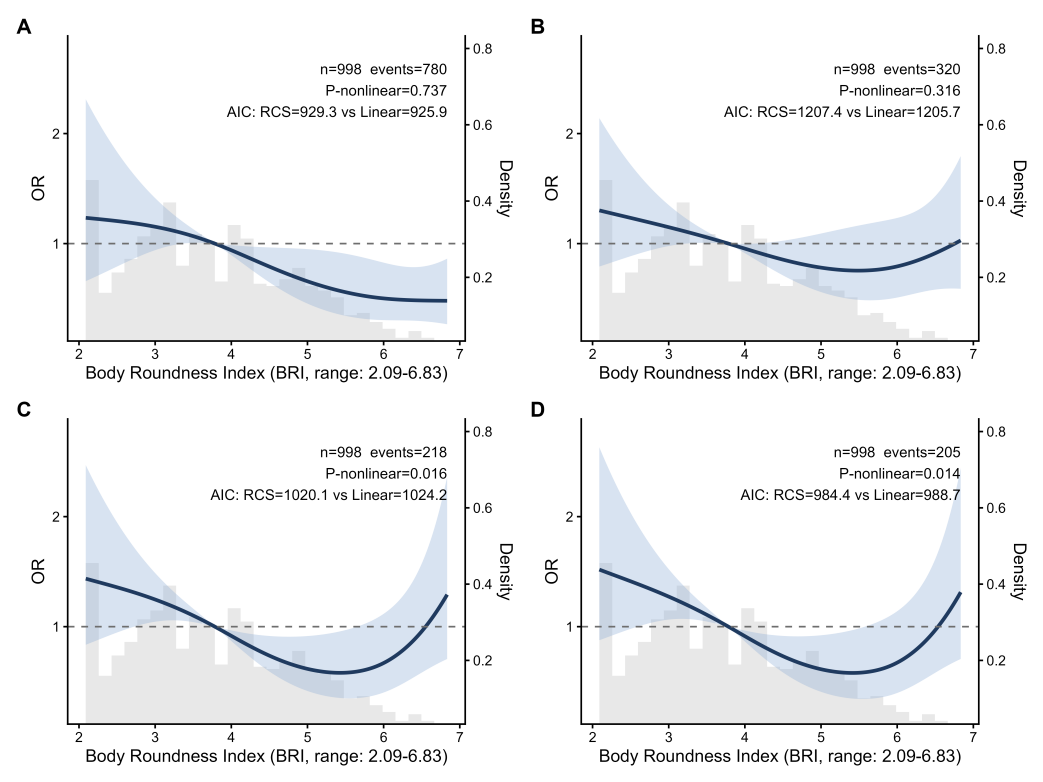


All adjusted for interventions, age, SBP, and DBP. Winsorization (5%) altered the tail shape. Together with wider confidence intervals and fewer events in the extreme range, this indicates that tail estimates are sensitive to limited information and tail-handling choices; therefore, associations at extreme BRI values should be interpreted cautiously, with inference primarily driven by the central range.

**eTable 6. Exploratory threshold analyses of BRI and Clinical pregnancy and Live birth.**

|  | OR (95% CI) | *P* |
| --- | --- | --- |
| Clinical pregnancy |  |  |
| Fitting by two-piecewise logistic-regression model |  |  |
| Inflection point | 5.28 |  |
| BRI<5.28 | 0.75(0.63, 0.88) | <0.001 |
| BRI>5.28 | 1.33(1.01, 1.73) | 0.043 |
| Log-likelihood ratio test |  | 0.004 |
| Live birth |  |  |
| Fitting by two-piecewise logistic-regression model |  |  |
| Inflection point | 5.20 |  |
| BRI<5.20 | 0.74(0.62, 0.88) | <0.001 |
| BRI>5.20 | 1.28(0.96, 1.68) | 0.073 |
| Log-likelihood ratio test |  | 0.006 |

All adjusted for interventions, age, SBP, and DBP.

**eTable 7. Stratify by BMI categories and interaction effects for the association between the BRI and reproductive outcomes.**

| Outcome | BMI categories | No./ Total No. | OR(95%CI), *P* | *P* for interaction |
| --- | --- | --- | --- | --- |
| ovulation |  |  |  | 0.271 |
|  | BMI < 24 kg/m² | 439/529 | 0.98 (0.75–1.27), P=0.884 |  |
|  | BMI ≥ 24 kg/m² | 341/469 | 0.82 (0.70–0.97), P=0.023 |  |
| conception |  |  |  | 0.804 |
|  | BMI < 24 kg/m² | 183/529 | 0.92 (0.75–1.14), P=0.448 |  |
|  | BMI ≥ 24 kg/m² | 137/469 | 0.95 (0.81–1.12), P=0.560 |  |
| Clinical pregnancy |  |  |  | 0.355 |
|  | BMI < 24 kg/m² | 133/529 | 0.88 (0.69–1.11), P=0.264 |  |
|  | BMI ≥ 24 kg/m² | 85/469 | 1.01 (0.84–1.21), P=0.943 |  |
| Live birth |  |  |  | 0.312 |
|  | BMI < 24 kg/m² | 125/529 | 0.83 (0.65–1.06), P=0.140 |  |
|  | BMI ≥ 24 kg/m² | 80/469 | 0.98 (0.81–1.18), P=0.805 |  |

All adjusted for interventions, age, SBP, and DBP.

**eFigure 6. Overlaps between BMI categories and BRI quartiles.**


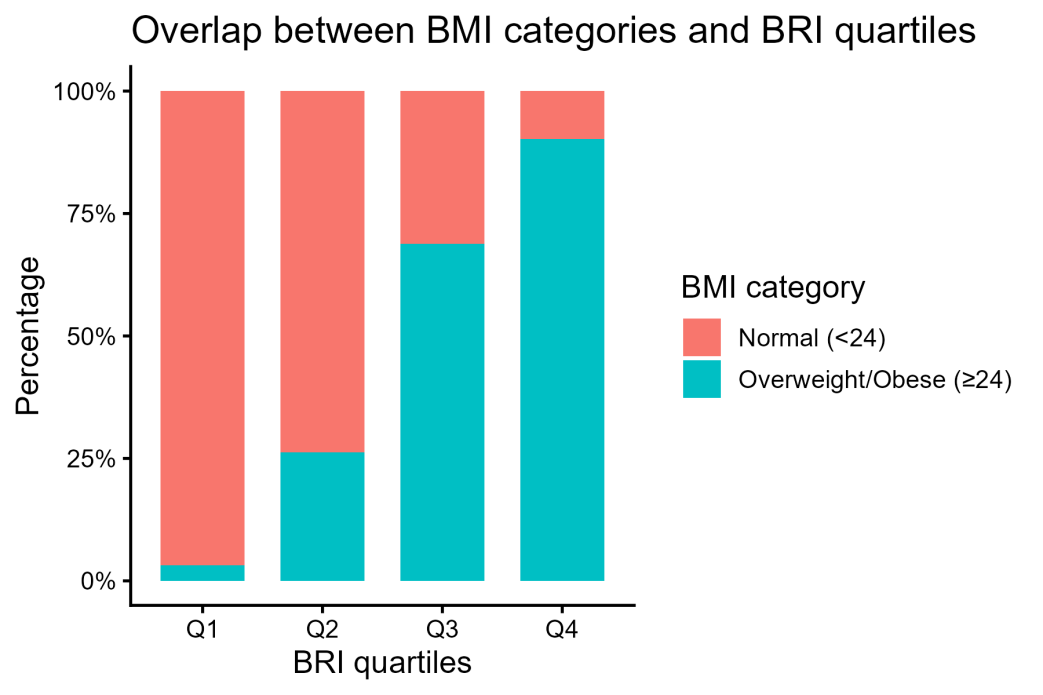


**eFigure 7. Distribution of BMI and BRI quartiles.**


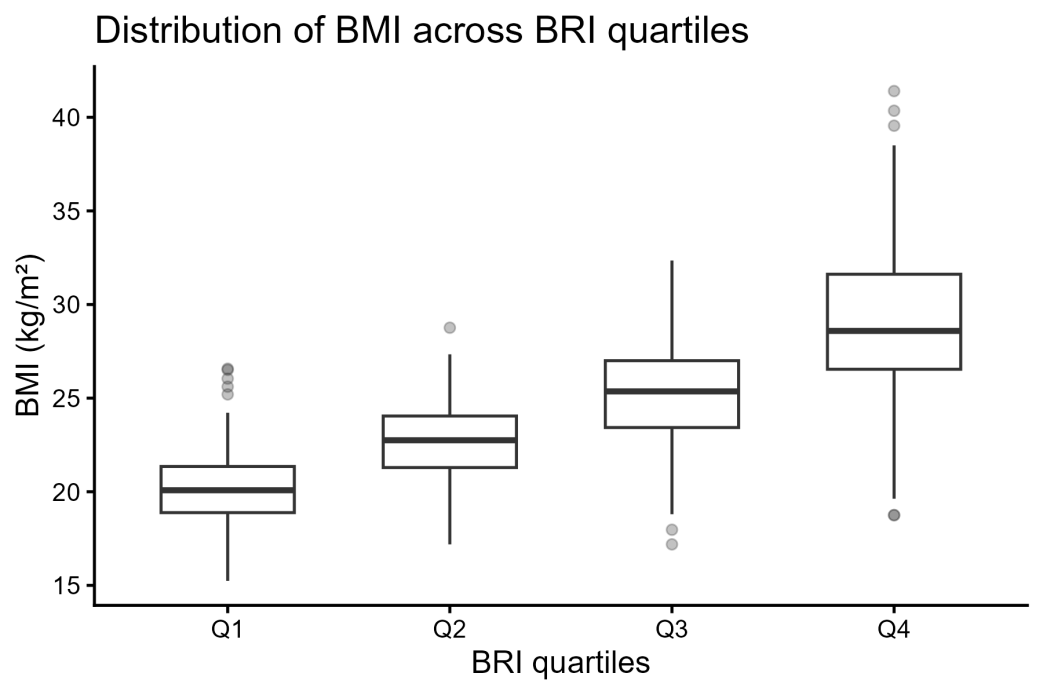

Supplement: Supplementary file 1 [file Table_1.DOCX]
